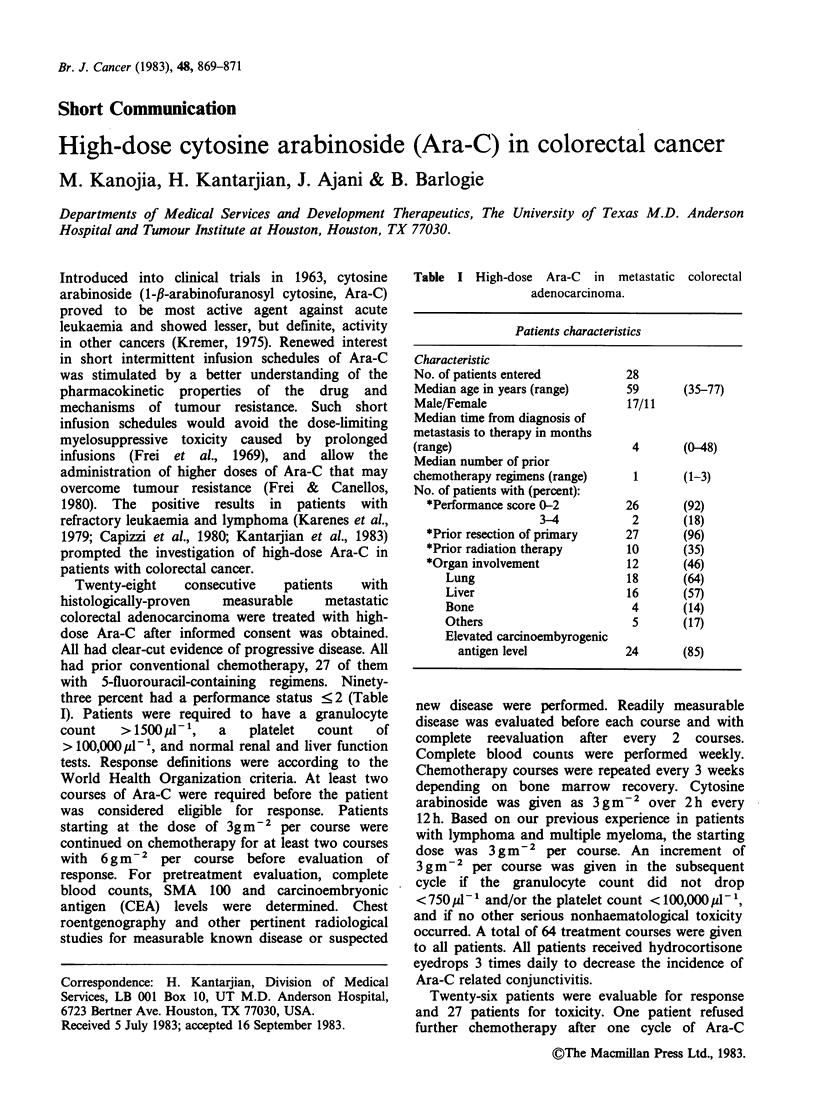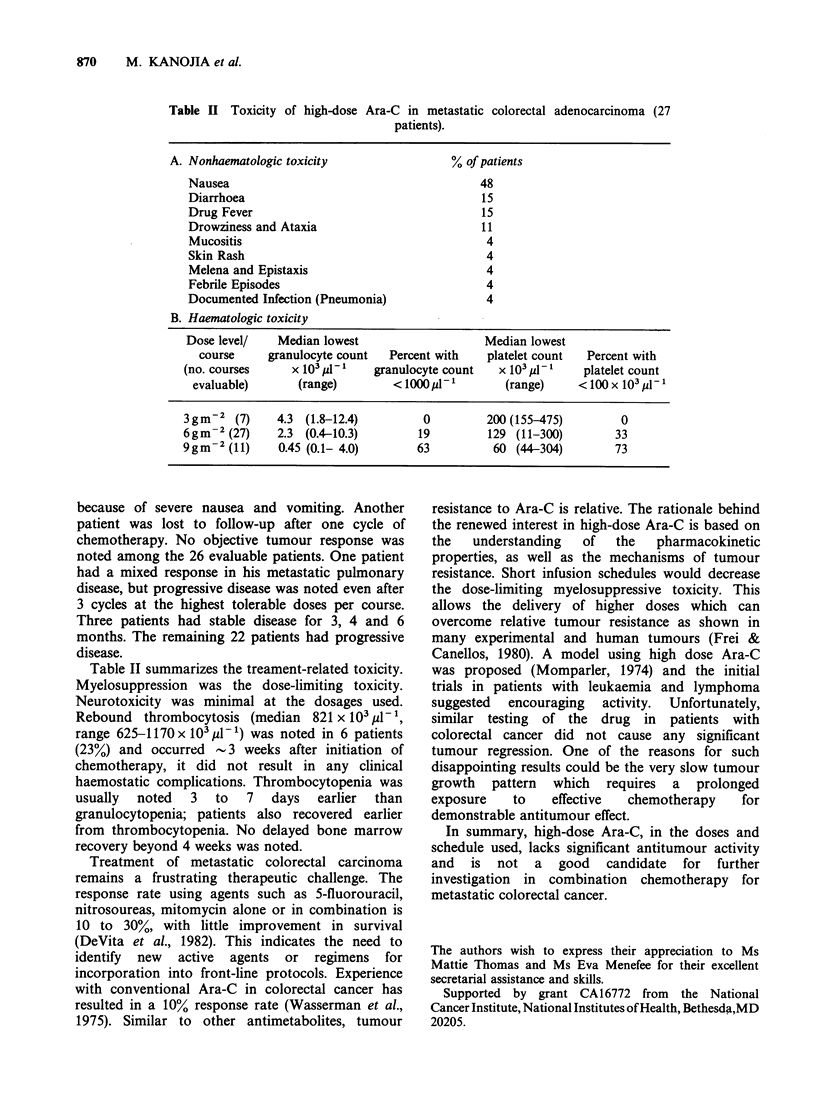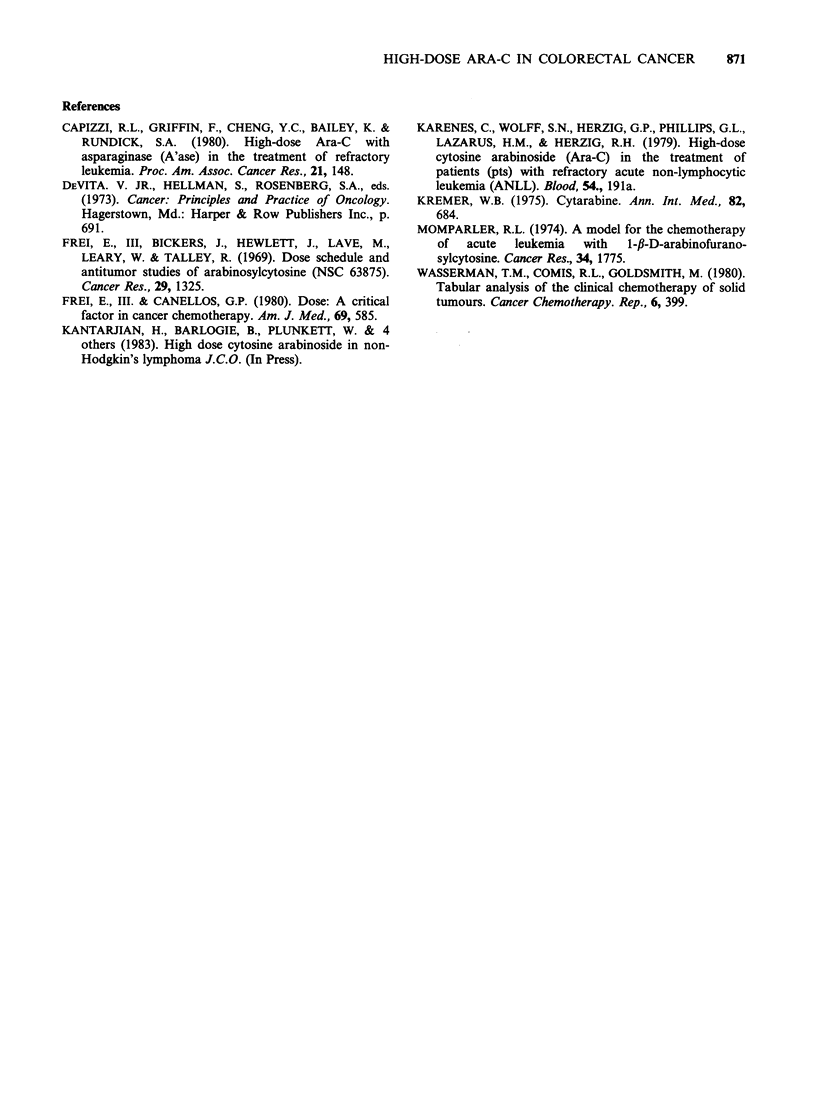# High-dose cytosine arabinoside (Ara-C) in colorectal cancer.

**DOI:** 10.1038/bjc.1983.280

**Published:** 1983-12

**Authors:** M. Kanojia, H. Kantarjian, J. Ajani, B. Barlogie


Br. J. Cancer (1983), 48, 869–871

### Short Communication

### High-dose cytosine arabinoside (Ara-C) in colorectal cancer

M. Kanojia, H. Kantarjian, J. Ajani & B. Barlogie

*Departments of Medical Services and Development Therapeutics, The University of Texas M.D. Anderson Hospital and Tumour Institute at Houston, Houston, TX 77030.*

Introduced into clinical trials in 1963, cytosine arabinoside (1-$\beta$-arabinofuranosyl cytosine, Ara-C) proved to be most active agent against acute leukaemia and showed lesser, but definite, activity in other cancers (Kremer, 1975). Renewed interest in short intermittent infusion schedules of Ara-C was stimulated by a better understanding of the pharmacokinetic properties of the drug and mechanisms of tumour resistance. Such short infusion schedules would avoid the dose-limiting myelosuppressive toxicity caused by prolonged infusions (Frei *et al.*, 1969), and allow the administration of higher doses of Ara-C that may overcome tumour resistance (Frei & Canellos, 1980). The positive results in patients with refractory leukaemia and lymphoma (Karenes *et al.*, 1979; Capizzi *et al.*, 1980; Kantarjian *et al.*, 1983) prompted the investigation of high-dose Ara-C in patients with colorectal cancer.

Twenty-eight consecutive patients with histologically-proven measurable metastatic colorectal adenocarcinoma were treated with high-dose Ara-C after informed consent was obtained. All had clear-cut evidence of progressive disease. All had prior conventional chemotherapy, 27 of them with 5-fluorouracil-containing regimens. Ninety-three percent had a performance status $\leq 2$ (Table I). Patients were required to have a granulocyte count $>1500\,\mu l^{-1}$, a platelet count of $>100{,}000\,\mu l^{-1}$, and normal renal and liver function tests. Response definitions were according to the World Health Organization criteria. At least two courses of Ara-C were required before the patient was considered eligible for response. Patients starting at the dose of $3 g m^{-2}$ per course were continued on chemotherapy for at least two courses with $6 g m^{-2}$ per course before evaluation of response. For pretreatment evaluation, complete blood counts, SMA 100 and carcinoembryonic antigen (CEA) levels were determined. Chest roentgenography and other pertinent radiological studies for measurable known disease or suspected new disease were performed. Readily measurable disease was evaluated before each course and with complete reevaluation after every 2 courses. Complete blood counts were performed weekly. Chemotherapy courses were repeated every 3 weeks depending on bone marrow recovery. Cytosine arabinoside was given as $3 g m^{-2}$ over 2 h every 12 h. Based on our previous experience in patients with lymphoma and multiple myeloma, the starting dose was $3 g m^{-2}$ per course. An increment of $3 g m^{-2}$ per course was given in the subsequent cycle if the granulocyte count did not drop $<750\,\mu l^{-1}$ and/or the platelet count $<100{,}000\,\mu l^{-1}$, and if no other serious nonhaematological toxicity occurred. A total of 64 treatment courses were given to all patients. All patients received hydrocortisone eyedrops 3 times daily to decrease the incidence of Ara-C related conjunctivitis.

Twenty-six patients were evaluable for response and 27 patients for toxicity. One patient refused further chemotherapy after one cycle of Ara-C

**Table I** High-dose Ara-C in metastatic colorectal adenocarcinoma.

| *Patients characteristics* | | |
|---|---|---|
| *Characteristic* | | |
| No. of patients entered | 28 | |
| Median age in years (range) | 59 | (35–77) |
| Male/Female | 17/11 | |
| Median time from diagnosis of metastasis to therapy in months (range) | 4 | (0–48) |
| Median number of prior chemotherapy regimens (range) | 1 | (1–3) |
| No. of patients with (percent): | | |
| *Performance score 0–2 | 26 | (92) |
| 3–4 | 2 | (18) |
| *Prior resection of primary | 27 | (96) |
| *Prior radiation therapy | 10 | (35) |
| *Organ involvement | 12 | (46) |
| Lung | 18 | (64) |
| Liver | 16 | (57) |
| Bone | 4 | (14) |
| Others | 5 | (17) |
| Elevated carcinoembyrogenic antigen level | 24 | (85) |

Correspondence: H. Kantarjian, Division of Medical Services, LB 001 Box 10, UT M.D. Anderson Hospital, 6723 Bertner Ave. Houston, TX 77030, USA.
Received 5 July 1983; accepted 16 September 1983.

©The Macmillan Press Ltd., 1983.

**Table II** Toxicity of high-dose Ara-C in metastatic colorectal adenocarcinoma (27 patients).

| A. *Nonhaematologic toxicity* | *% of patients* |
|---|---|
| Nausea | 48 |
| Diarrhoea | 15 |
| Drug Fever | 15 |
| Drowziness and Ataxia | 11 |
| Mucositis | 4 |
| Skin Rash | 4 |
| Melena and Epistaxis | 4 |
| Febrile Episodes | 4 |
| Documented Infection (Pneumonia) | 4 |

B. *Haematologic toxicity*

| Dose level/ course (no. courses evaluable) | Median lowest granulocyte count $\times 10^3 \mu l^{-1}$ (range) | Percent with granulocyte count $< 1000 \mu l^{-1}$ | Median lowest platelet count $\times 10^3 \mu l^{-1}$ (range) | Percent with platelet count $< 100 \times 10^3 \mu l^{-1}$ |
|---|---|---|---|---|
| $3 g m^{-2}$ (7) | 4.3 (1.8–12.4) | 0 | 200 (155–475) | 0 |
| $6 g m^{-2}$ (27) | 2.3 (0.4–10.3) | 19 | 129 (11–300) | 33 |
| $9 g m^{-2}$ (11) | 0.45 (0.1– 4.0) | 63 | 60 (44–304) | 73 |

because of severe nausea and vomiting. Another patient was lost to follow-up after one cycle of chemotherapy. No objective tumour response was noted among the 26 evaluable patients. One patient had a mixed response in his metastatic pulmonary disease, but progressive disease was noted even after 3 cycles at the highest tolerable doses per course. Three patients had stable disease for 3, 4 and 6 months. The remaining 22 patients had progressive disease.

Table II summarizes the treament-related toxicity. Myelosuppression was the dose-limiting toxicity. Neurotoxicity was minimal at the dosages used. Rebound thrombocytosis (median $821 \times 10^3 \mu l^{-1}$, range $625–1170 \times 10^3 \mu l^{-1}$) was noted in 6 patients (23%) and occurred ~3 weeks after initiation of chemotherapy, it did not result in any clinical haemostatic complications. Thrombocytopenia was usually noted 3 to 7 days earlier than granulocytopenia; patients also recovered earlier from thrombocytopenia. No delayed bone marrow recovery beyond 4 weeks was noted.

Treatment of metastatic colorectal carcinoma remains a frustrating therapeutic challenge. The response rate using agents such as 5-fluorouracil, nitrosoureas, mitomycin alone or in combination is 10 to 30%, with little improvement in survival (DeVita *et al.*, 1982). This indicates the need to identify new active agents or regimens for incorporation into front-line protocols. Experience with conventional Ara-C in colorectal cancer has resulted in a 10% response rate (Wasserman *et al.*, 1975). Similar to other antimetabolites, tumour resistance to Ara-C is relative. The rationale behind the renewed interest in high-dose Ara-C is based on the understanding of the pharmacokinetic properties, as well as the mechanisms of tumour resistance. Short infusion schedules would decrease the dose-limiting myelosuppressive toxicity. This allows the delivery of higher doses which can overcome relative tumour resistance as shown in many experimental and human tumours (Frei & Canellos, 1980). A model using high dose Ara-C was proposed (Momparler, 1974) and the initial trials in patients with leukaemia and lymphoma suggested encouraging activity. Unfortunately, similar testing of the drug in patients with colorectal cancer did not cause any significant tumour regression. One of the reasons for such disappointing results could be the very slow tumour growth pattern which requires a prolonged exposure to effective chemotherapy for demonstrable antitumour effect.

In summary, high-dose Ara-C, in the doses and schedule used, lacks significant antitumour activity and is not a good candidate for further investigation in combination chemotherapy for metastatic colorectal cancer.

The authors wish to express their appreciation to Ms Mattie Thomas and Ms Eva Menefee for their excellent secretarial assistance and skills.

Supported by grant CA16772 from the National Cancer Institute, National Institutes of Health, Bethesda, MD 20205.

**References**

CAPIZZI, R.L., GRIFFIN, F., CHENG, Y.C., BAILEY, K. & RUNDICK, S.A. (1980). High-dose Ara-C with asparaginase (A'ase) in the treatment of refractory leukemia. *Proc. Am. Assoc. Cancer Res.*, **21**, 148.

DEVITA. V. JR., HELLMAN, S., ROSENBERG, S.A., eds. (1973). *Cancer: Principles and Practice of Oncology*. Hagerstown, Md.: Harper & Row Publishers Inc., p. 691.

FREI, E., III, BICKERS, J., HEWLETT, J., LAVE, M., LEARY, W. & TALLEY, R. (1969). Dose schedule and antitumor studies of arabinosylcytosine (NSC 63875). *Cancer Res.*, **29**, 1325.

FREI, E., III. & CANELLOS, G.P. (1980). Dose: A critical factor in cancer chemotherapy. *Am. J. Med.*, **69**, 585.

KANTARJIAN, H., BARLOGIE, B., PLUNKETT, W. & 4 others (1983). High dose cytosine arabinoside in non-Hodgkin's lymphoma *J.C.O.* (In Press).

KARENES, C., WOLFF, S.N., HERZIG, G.P., PHILLIPS, G.L., LAZARUS, H.M., & HERZIG, R.H. (1979). High-dose cytosine arabinoside (Ara-C) in the treatment of patients (pts) with refractory acute non-lymphocytic leukemia (ANLL). *Blood*, **54.**, 191a.

KREMER, W.B. (1975). Cytarabine. *Ann. Int. Med.*, **82,** 684.

MOMPARLER, R.L. (1974). A model for the chemotherapy of acute leukemia with 1-β-D-arabinofuranosylcytosine. *Cancer Res.*, **34,** 1775.

WASSERMAN, T.M., COMIS, R.L., GOLDSMITH, M. (1980). Tabular analysis of the clinical chemotherapy of solid tumours. *Cancer Chemotherapy. Rep.*, **6,** 399.